# Stepping towards health: a scoping review of square-stepping exercise protocols and outcomes in older adults

**DOI:** 10.1186/s12877-024-05187-8

**Published:** 2024-07-10

**Authors:** Vitor A. A. A. Siqueira, Ryosuke Shigematsu, Emerson Sebastião

**Affiliations:** 1 Department of Health and Kinesiology, University of Illinois at Urbana- Champaign, Urbana, IL USA; 2 Department of Health and Sports Sciences, Chukyo University, Toyota, Japan; 3 Department Health and Kinesiology, University of Illinois Urbana-Champaign, Urbana, IL USA

**Keywords:** Cognition, Functional rehabilitation, Motor function, Neurological disease

## Abstract

**Background:**

Square-Stepping Exercise (SSE) is a type of physical-cognitive exercise. Such exercise has been used as an exercise approach in different studies with older adults. This scoping review provides an overview of the protocols and outcomes of studies employing the SSE in older adults.

**Methods:**

We searched in the PubMed, CINAHL, Scopus, CABI Global Health, and Web of Science databases for articles published between 2006 (first research article published on SSE) to December 2023 that met a robust inclusion criterion. The search yielded 424 articles, and after inclusion criteria being applied, 37 articles were included in the final analysis.

**Results:**

A total of 37 studies were included in the final analysis. Thirty-three out of the 37 studies focused on apparently healthy older adults, while four were conducted with older adults with neurological disease (i.e., multiple sclerosis, Parkinson’s disease, and stroke). Most studies (*n* = 25) adopted an experiment (i.e., randomized controlled trial) or quasi-experimental approach, while 12 were classified as non-randomized (i.e., cross-sectional, mixed methods). The studies were conducted in different parts of the globe and adopted three major formats of intervention delivery, namely in-person, online, and home-based. Frequency, SSE session duration and intervention length significantly varied among studies, and reported outcomes were in the domains of physical and cognitive function.

**Conclusion:**

This review comprehensively described the characteristics of 37 studies employing SSE in apparently healthy older adults and older adults with neurological diseases. The findings demonstrated that SSE has been used by researchers across the globe, adopting a variety of forms of delivery, and to particularly improve physical and cognitive function of different segments of the older adult population. The review further identified important gaps in research, including the restricted outcomes, and the lack of studies combining SSE with more traditional exercise modalities to address potential combinatory effects.

**Supplementary Information:**

The online version contains supplementary material available at 10.1186/s12877-024-05187-8.

## Introduction

Population aging is a phenomenon occurring across the globe, with estimates from the World Health Organization indicating that by 2030, one in six individuals in the world will be aged 60 years or older [1]. However, despite the increased life expectancy, the added years are not accompanied by increased health [1]. Aging has long been recognized as a main risk factor for major debilitating and life-threatening conditions, including cancer, cardiovascular and neurodegenerative diseases. These conditions, profoundly impact independence, and quality of life of older adults [2–4].

Physical activity, particularly exercise training has been highly recommended for older adults, even in the presence of chronic diseases and conditions [5, 6]. Regular exercise participation has the potential to prevent or delay the onset of a wide range of chronic diseases and conditions common in old ages, as well as counteract most of the physiological, functional, and psychological declines associated with aging [6–10]. Additionally, exercise training has been recommended to improve physical performance (e.g., muscle strength, cardiorespiratory fitness) and cognitive function (e.g., global cognitive and executive function) in older adults [6, 11–15].

Current physical activity guidelines recommend that older adults engage in at least ≥ 150 min of moderate-intensity aerobic exercise or ≥ 75 min of vigorous-intensity aerobic exercise per week, or an equivalent combination of the two [16]. Additionally, existing recommendations emphasize the importance of exercise training and cognitive stimulation in older adult care [17]. This is reinforced by a recent systematic review in which researchers observed that combined exercise and cognitive training program resulted in better physical and cognitive function performance compared to single-domain training [18]. Thus, exercise programs employing a motor-cognitive approach are important for this population.

As a type of motor-cognitive training, the Square-Stepping Exercise (SSE) holds promise as an effective program/intervention for improving physical and cognitive function in older adults. Since its development by Japanese researchers in 2006 to be a new exercise modality focusing on vital and psychosocial functions (i.e., fall prevention, improved physical and cognitive function) [19], SSE has been effectively implemented in different forms, settings, and populations. However, despite its potential benefits, there remains a lack of comprehensive understanding regarding the characteristics of SSE programs employed in various populations of older adults – including those with neurological conditions. This is important because SSE is an easy-to-perform, low cost, and highly accessible exercise modality that could be implemented and scalable in community-based exercise programs and as home-based exercise, minimizing barriers associated with accessibility of exercise programs available for the older adult population. This is further important because SSE may further be employed in combination with more traditional exercise modalities in a multicomponent exercise program. By conducting a scoping review, this study aims to fill this gap in the literature by providing a systematic overview of SSE interventions conducted in apparently healthy older adults and those with neurological conditions. In addition, the findings of this review hold significant implications for the development of effective exercise interventions tailored to the needs of older adults, especially those with neurological conditions. To this end, by comprehensively summarizing the characteristics of SSE programs and outcomes reported in the literature, this scoping review seeks to help inform the design and implementation of future exercise interventions aimed at improving not only physical and cognitive function, but health and well-being of older adults. Furthermore, identifying research gaps and highlighting opportunities for further investigation will contribute to advancing our understanding of the role of SSE in promoting healthy aging and managing neurological conditions.

Despite recent systematic reviews with meta-analysis focusing on the effectiveness of SSE for physical and cognitive function, as well as fall prevention in older adults [20, 21], these studies have not provided a comprehensive overview of SSE protocols and outcomes due to their restricted inclusion criteria and narrow research questions. Therefore, this scoping review sought to fill this gap by identifying studies throughout the world that utilized SSE in older adults. The review will examine the characteristics of the protocols employed as well as the outcomes investigated. This scoping review aims to address two primary questions:

1. What are the characteristics (e.g., frequency, duration, delivery mode, targeted population) and aims of SSE interventions conducted in apparently healthy older adults and older adults with neurological conditions?
2. What type(s) of outcomes have been reported?

By systematically analyzing the literature, this review not only provides an overview and insights into SSE studies but also identifies research gaps and highlight opportunities for further research.

## Materials and methods

### Nature of review

The present scope review was carried out according to systematic principles found at the Preferred Reporting Items of Systematic Review and Meta-Analyzes-PRISMA [22, 23]. Despite sharing common methodologies and characteristics with systematic reviews, scoping reviews are different from systematic reviews in both purpose and aims [24]. Scoping reviews are designed to address broader research questions, offering an overview of available research evidence without centering on a specific research question. Therefore, a scoping review was chosen for this study [24–26].

### Inclusion and exclusion criteria

The article search was conducted during the spring, summer and fall 2023 semesters, and the selection criteria were developed using the PICOS (Participants, Intervention, Context, Outcome, and Study design) framework [27]. The following inclusion criteria was employed: (a) Study design: randomized controlled trials, quasi-experimental, pre-post, single group, cross-sectional; (b) geographical location: global; (c) participants: apparently healthy older adults (i.e., 60 years and over) and/or patients with neurological conditions; (d) intervention: studies using SSE as primary or comparative exercise modality; (e) outcome: any; (f) article type: original peer-reviewed publications; and (g) language: English. Studies were excluded if they met any of the following criteria: (a) stepping interventions that did not followed the original SSE, (b) SSE intervention being delivered in young adults (c) non-English articles, (d) non-peer-reviewed publications, (e) non-empirical research, and (f) reviews, commentaries, study protocols, editorials, or abstracts.

### Search strategy

A comprehensive search was conducted by a librarian trained graduate student in major electronic bibliographic databases: PubMed, CINAHL, Scopus, CABI Global Health, and Web of Science. In addition, the “grey” literature was consulted through Google Scholar. The search term included “square-stepping exercise”, “square stepping exercise”, “square-stepping exercise” AND “older adults”, “square-stepping exercise” AND “elderly”. Two authors reviewed the articles for title and abstract against the study selection criteria independently and retrieved potential articles for full-text evaluation. Afterward, a cited reference search (e.g., forward reference search) and reference list search (e.g., backward reference search) were conducted and repeated to verify the availability of any additional articles. Two authors jointly determined the articles to be included in the scoping review after full-text reading. Any discordance was settled by face-to-face meeting or with the help of a third reviewer if needed.

### Data extraction and synthesis

A standardized form was created for data extraction to record the following information from the included studies: ***(1) study information***: first author, year of publication, objectives, study design, sample details, location (Table 1); ***(2) SSE intervention characteristics***: age of participants, delivery mode (e.g., in-person, online, home-based), protocol details, study duration, and primary outcomes (Table 2) and; ***(3) main findings***: summary of key results and conclusion (Table 3). Data extraction was performed independently by two researchers to ensure accuracy and consistency. Discrepancies were resolved through discussion, and, if necessary, consultation with a third researcher with significant experience in conducting literature reviews and who were not part of the author’s block. To synthesize the data from the included studies, we employed descriptive statistics. We used descripted statistics to describe and summarize common aspects of the included SSE studies. The synthesis process involved the following steps to describe common aspects of the SSE studies such as study design, participant demographics, intervention characteristics, and outcomes. Frequencies and percentages were calculated to provide a quantitative overview of the included studies.

## Results

### Study selection

**Fig. 1:**
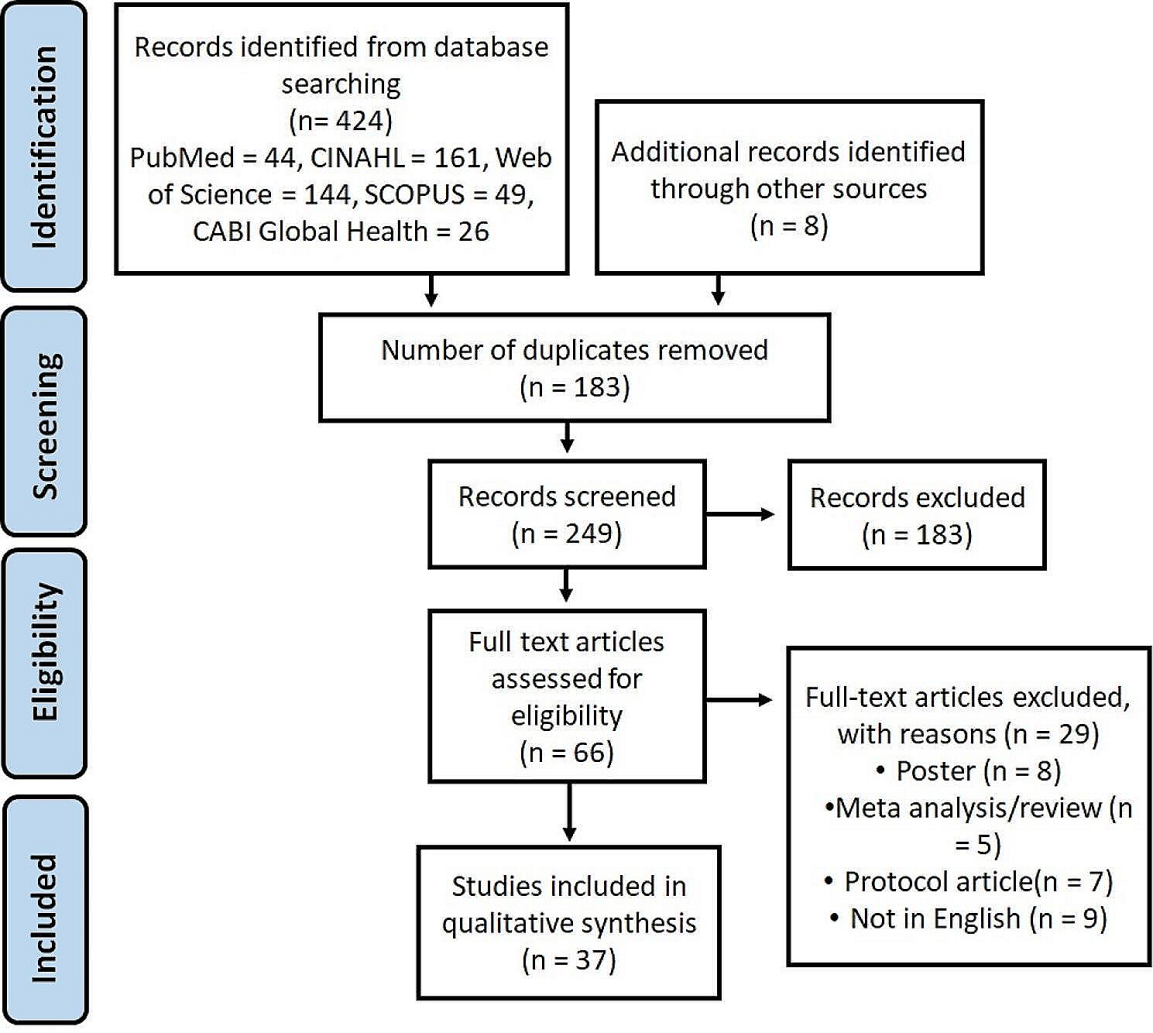
PRISMA flow diagram for study selection Note: PRISMA = preferred reporting items for systematic reviews and meta-analysis; CHW = community health workers

A detailed screening process for the eligible studies has been provided as a flowchart in Fig. 1. A total of 424 articles were identified from the databases and 8 through backward reference search. All articles were later imported to Mendeley Reference Management Software. After removing duplicates, 249 articles were reviewed by title and abstract; 66 articles were selected for full-text review. One included article was not freely available through the institutional library databases. Thus, one of the authors of the referred article was contacted via email and promptly responded with a digital copy (i.e., pdf). After a thorough assessment, 29 articles were excluded in accordance with the exclusion criteria, and the reasons for exclusion are displayed in Fig. 1. Finally, 37 articles were selected to be included in this review [19, 28–62].

### Basic characteristics of included studies

**Fig. 2:**
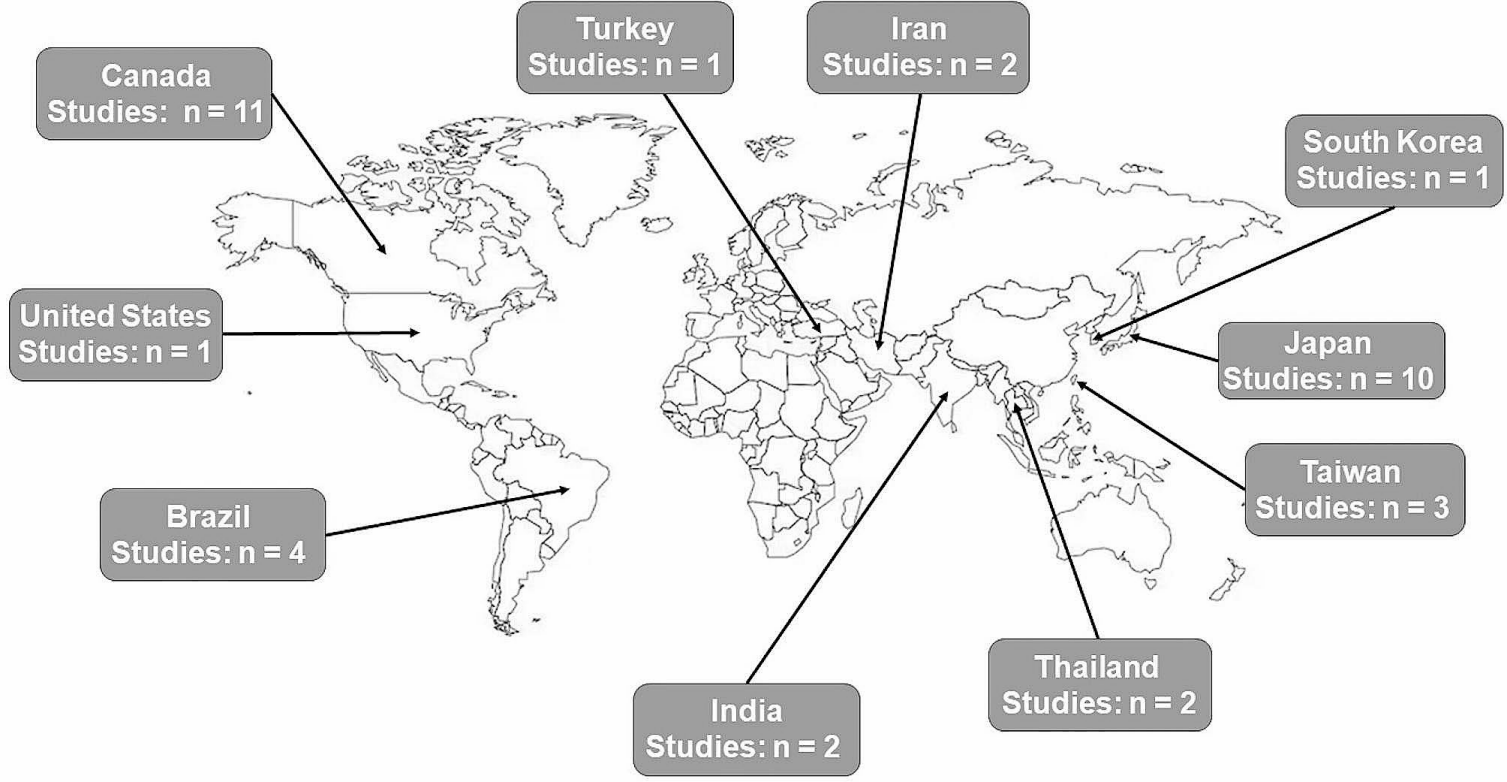
Number of square-stepping exercise studies separated by country

Table 1 describes the basic characteristics of the studies and their participants. All 37 studies included were published between 2006 and 2023. Among these, 24 were randomized control trials (RCT), and 13 were non-RCT (i.e., quasi-experimental, mixed-methods, and cross-sectional). The studies were conducted globally, with nearly 57% of them (*n* = 21). Figure 2 provides in detail the number of SSE studies separated by country. Of the 37 studies, 33 recruited and enrolled apparently healthy older adults, while four were conducted in older adults with neurological conditions (i.e., multiple sclerosis, Parkinson’s disease, and stroke). The number of participants in the included studies ranged from 10 to 4005, with females comprising between 50 and 100% of the studies’ sample. Most studies shared a common goal of improving physical or cognitive function in their respective enrolled populations, with a small portion investigating primarily feasibility metrics (e.g., safety, acceptability).

**Table 1:** Sample details and study design of the 37 included studies

Author	Location	Sample details	Objective	Study design
Shigematsu et al. [19]	Japan	Older adults *N* = 52	Test whether our new exercise program (a Square-Stepping Exercise: SSE) would improve lower-extremity functional fitness in older adults.	Non-RCT
Shigematsu et al. [54]	Japan	Older adults *N* = 68	To compare two exercise programs—Square-Stepping Exercise (SSE) and walking—for improving the fitness of the lower extremities.	RCT
Shigematsu et al. [56]	Japan	Older adults *N* = 39	To compare the effects of Square-Stepping Exercise (SSE) training, with strength and balance training.	RCT
Shigematsu et al. [55]	Japan	Older adults *N* = 68	Assess functional fitness and adherence to the SSE program among older adults persons in a long term, observational setting.	RCT
Teixeira et al. [57]	Brazil	Older adults *N* = 41	To analyze the effect of 16 weeks of SSE on cognitive functions in non-demented community-dwelling older people.	Non-RCT
Teixeira et al. [58]	Brazil	Older adults *N* = 86	To analyze the effect of a program using Square-Stepping Exercises sequences only on functional fitness in older adults.	Non-RCT
Pereira et al. [46]	Brazil	Older adults *N* = 32	To analyze the effects of Square-Stepping Exercise on depressive symptoms, balance, and functional mobility in older adults	Non-RCT
Túbero et al. [60]	Brazil	Older adults with stroke *N* = 13	To verify the effects of Square-Stepping Exercise on cognitive functions, depressive symptoms and the body balance of cerebral stroke sequel patients.	Non-RCT
Gill et al. [36]	Canada	Older adults *N* = 44	To determine the impact of group-based exercise and dual-task training on gait and vascular health, in active community-dwelling older adults without dementia.	RCT
Gregory et al. [35]	Canada	Older adults *N* = 44	To examine the effect of a group-based standard exercise program for older adults, with and without dual-task training, on cognitive function in older adults without dementia.	RCT
Jindo et al. [38]	Japan	Older adults *N* = 35	To investigate how goal setting aimed at increasing daily physical activity by 1,000 steps per day, influenced lower-extremity physical function during an SSE exercise program.	Non-RCT
Jindo et al. [39]	Japan	Older adults *N* = 68	To compare the effect on lower-extremity physical function between an exercise intervention with and without the use of a pedometer.	Non-RCT
Jindo et al. [62].	Japan	Older adults *N* = 46	To investigate how daily life Physical activity modulates the effects of an exercise program on lower-extremity physical function in older adults.	Non-RCT
Ravichandran et al. [63]	India	Older adults with Parkinson’s disease *N* = 37	Analyze the effects of square‑stepping exercise among Parkinson’s disease patients in terms of improving balance and reducing fall risk.	RCT
Heath et al. [33]	Canada	Older adults *N* = 63	To determine whether a cognitive-based visuospatial stepping task included in a multiple-modality exercise training program renders an additive post intervention benefit in executive control for persons Self-reporting a cognitive complaint.	RCT
Shellington et al. [51]	Canada	Older adults *N* = 19	To determine the feasibility and utility of the HealtheBrain smartphone app to deliver SSE outside the laboratory among older adults with and without cognitive impairment.	Non-RCT
Chang et al. [34]	China	Older adults *N* = 102	To determine whether a synergistic exercise model based on aerobics with additional fall-preventive components could provide extra benefits compared with the same duration of aerobic-synergistic exercise alone.	Non-RCT
Boa Sorte Silva et al. [29]	Canada	Older adults *N* = 127	To explore the influence of a 24-week multiple-modality exercise program associated with a mind-motor training in cardiovascular health and fitness in community-dwelling older adults, compared to multiple-modality exercise alone.	RCT
Boa Sorte Silva et al. [32]	Canada	Older adults *N* = 127	To investigate the effects of multiple-modality exercise with or without additional mind-motor training on mobility outcomes in older adults with subjective cognitive complaints.	RCT
Boa Sorte Silva et al. [30]	Canada	Older adults *N* = 127	To investigate the effects of group-based based, multiple-modality exercise with additional SSE would improve cognition when compared to multiple-modality exercise alone in older adults with SCC living in the community.	RCT
Shellington et al. [52]	Canada	Older adults *N* = 25	To determine the feasibility of SSE in adults with type 2 diabetes and self-reported cognitive complaints.	RCT
Shellington et al. [53]	Canada	Older adults *N* = 22	To determine if an SSE program is feasible in an older adult population with knee OA, assessed via recruitment and attendance.	RCT
Sebastião et al. [50]	United States	Older adults with multiple sclerosis *N* = 25	Test the feasibility, acceptability, and possible efficacy of a home-based SSE intervention for older adults with MS.	RCT
Boa Sorte Silva et al. [31]	Canada	Older adults with risk of dementia *N* = 127	To explore memory function and task-related to brain FC changes following multiple-modality exercise and mind–motor training in older adults with subjective cognitive complaints.	RCT
Sawasdee et al. [48]	Thailand	Older adults *N* = 43	To evaluate the effects of modified Square-Stepping Exercise training on heart rate variability and body fat in older adults.	RCT
Uchida et al. [61]	Japan	Older adults *N* = 12	To examine the exercise intensity of Square-Stepping Exercise in community-dwelling late female older adults.	Non-RCT
Kim et al. [42]	Japan	Older adults *N* = 26	To investigate the effect of combined intake of CIT and LEU accompanied by exercise for 20 weeks on body composition, physical activity, and amino acid concentrations in older Japanese women with low body mass index using a randomized, double-blind, placebo-controlled design.	RCT
Kocaman et al. [41]	Japan	Older adults *N* = 30	To examine factors related to older adults’ participation in certain types of volunteer-managed preventive care exercises by focusing on the distance to exercise facilities and interpersonal social networks.	RCT
Sharma et al. [49]	India	Older adults *N* = 40	To compare the effects of cognitive and mind-motor training on cognition and functional skills in a community-dwelling sample of older adults.	RCT
Soma et al. [28]	Turkey	Older adults *N* = 4005	To investigate the effects of different exercise trainings on functionality in older fallers.	Non-RCT
Sow et al. [59]	China	Older adults *N* = 14	To examine the feasibility and effectiveness of SSE on executive function and gait variability among community-dwelling older adults.	RCT
Kraiwong et al. [43]	Thailand	Older adults *N* = 37	To explore the effects of group-based physical-cognitive trainings on physical and psychological outcomes among older adults with type 2 diabetes mellitus and balance impairment.	RCT
Liu et al. [45]	China	Older adults with Parkinson’s disease *N* = 28	To investigate the effects of SSE on cognitive function, especially executive function in people with Parkinson’s disease.	RCT
Cha et al. [37]	Korea	Older adults *N* = 20	To examine the effect of a Square-Stepping Exercise program on fall-related fitness and brain-derived neurotrophic factor levels.	RCT
Lees et al. [44]	Canada	Older adults *N* = 10	To investigate the effectiveness of Square-Stepping Exercises in addressing exercise barriers, increasing physical activity levels, and understanding the relationship between participant characteristics and post-intervention outcomes.	Non-RCT
Khalaji et al. [40]	Iran	Older adults *N* = 24	To examine whether consecutive implementation of enhanced expectancies, autonomy support, and external focus during practice would enhance the learning of a square-stepping task in older adults.	RCT
Sadeghian et al. [47]	Iran	Older adults *N* = 36	Compare the effects of Square-Stepping Exercise versus Tai Chi Chuan on functional fitness and fear of falling in older women aged 60 years and above.	RCT

Note: RCT – Randomized controlled trial; non-RCT – Nonrandomized controlled trial; SCC – subjective cognitive complaints; OA – Older adults; FC -brain functional connectivity; CIT- citrulline; LEU – leucine. Non-RCT: studies included quasi-experimental; cross-sectional observational and mixed-methods intervention design

### Characteristics of SSE interventions

Table 2 displays the characteristics of all SSE studies included in the present study. Thirty-four out of the 37 studies conducted an intervention using SSE [19, 29–50, 52–60, 62, 63], while three studies adopted a cross-sectional design approach [28, 51, 61]. To this end, the research protocols of the studies exhibited a broad spectrum as far as frequency and SSE session duration. For instance, the frequency of the studies involving intervention ranged from one to seven times per week, with sessions varying from 45 to 90 min each. The prevailing intervention frequency, observed in 50% (*n* = 17) of the articles that adopted intervention was three sessions of SSE per week. The average session duration accounting for warm-up and cooldown was found to be 90 min per session with some studies reporting no warm-up or cooldown component during the sessions. In terms of intervention duration (i.e., intervention length) studies last from one single day to 48 weeks, with 44% of the articles (*n* = 15) adopting an intervention duration between 12 and 24 weeks. Further, studies adopted three different methods to deliver the SSE intervention, which included in-person, home-based, and online. Most of the studies (*n* = 33) featured in-person interventions, with one study conducted in older adults with multiple sclerosis adopting a hybrid approach (i.e., limited laboratory visit followed by home-based practice) [50], and three (8%) opting for either home-based or online formats utilizing smartphone applications or video call [41, 44, 51]. In terms of outcomes, a significant portion of the studies (68%; *n* = 25) centered around physical function (e.g., mobility, balance) and cognitive function (e.g., memory) outcomes. Twelve studies (31.5%) explored diverse aspects (i.e., body composition, nutrition, exercise intensity, psychological aspects, and delivery and participation), with four (10.5%) of them specifically investigating feasibility metrics within SSE.

**Table 2:** Methodological characteristics of the 37 included studies investigating the square-stepping exercise as intervention

Author	Age Mean ± SD or Range	Intervention type and delivery mode	Intervention protocol	Intervention Duration, week	Primary outcomes & Secondary outcomes
Shigematsu et al. [19]	67.9 ± 5.6	In person	1x week 60-minute session (+ 10 min warm-up/10 min cooldown)	24	Supine-to-stand test, chair-stand in 10 s, walking round two cones, sit-and-reach, single leg balance with eyes closed
Shigematsu et al. [54]	68.6 ± 2.4	In person	2x week 70-minute session (15 min warm-up/15 min cooldown)	12	Functional fitness, self-reported scales, fall incidence
Shigematsu et al. [56]	69.1 ± 2.8	In person	2x week 40 min session (+ 15 min warm-up/15 min cooldown)	12	Strength, Balance, agility, reaction.
Shigematsu et al. [55]	69.1 ± 2.7	Home-based	-	1–4 years	Fall incidence and adherence
Teixeira et al. [57]	68.1 ± 7.5	In person	3 x week 40-minute session	16	Flexibility, agility, balance, aerobic endurance, motor coordination, upper limb strength resistance, berg scale, TUG
Teixeira et al. [58]	67.5 ± 6.5	In person	3 x week 40-minute session	16	Digital span test, Toulouse-Pieron concentrated attention test, modified card sorting test.
Pereira et al. [46]	76.3 ± 8.8	In person	2 x week 60-minute sessions	16	MMSE, GDS-15, TUG, 14-item Berg balance scale
Tubero et al. [60]	62.5 ± 8.3	In person	3x week 90-minute session	8	Mini mental state examination, brief cognitive screening battery, geriatric depression scale, Berg balance scale, TUG
Gill et al. [36]	73.5 ± 7.2	In person	2 or 3 x week minimum of 50 min session and maximum of 75.	26	Cognitive function, executive function, mental flexibility, processing speed, verbal learning and memory
Gregory et al. [35]	73.5 ± 7.2	In person	2 or 3 x week minimum of 50 min session and maximum of 75.	26	Mobility outcomes, vascular health
Jindo et al. [38]	68.9 ± 3.8	In person	1 x week 90 min session	9	Balance, STS, TUG, 5-m habitual walk, choice stepping reaction time, 6 min walk
Jindo et al. [39]	70.0 ± 3.6	In person	1 x week 30 to 40 min sessions (15 min warm-up, 20 min a recreational activity and 15 min cooldown)	11	Single -leg balance, five-repetition sit to stand, 5-m habitual walk, choice stepping reaction time
Jindo et al. [62].	70.1 ± 3.5	In person	1 x week 90-minute session (15 min warm-up, 40 min of SSE, 20 min a recreational activity and 15 min cooldown)	11	Single leg balance, Habitual walking speed, choice-stepping reaction time, TUG, five-repetition sit to stand
Ravichandran et al. [63]	68.8 (60–70)	In person	5x week	4	Berg balance scale, TUG
Heath et al. [33]	67.0 ± 7.3	In person	3x week 60-minute session (5-minute warm-up/ 20 min moderate-to-vigorous intensity aerobic training/5 -minute cooldown/ 10-minute resistance training/15-minute balance training/5-minute stretching)	24	Oculomotor processing
Shellington et al. [51]	68.3 ± 5.4	Online - app based	1–7 x week	48	Feasibility, recruitment, utility of the app
Chang et al. [34]	76.1 ± 4.6	In person	2x week 40 min session (+ 10 min warm-up/10 min cooldown)	12	Aerobic endurance, leg strength, flexibility, reaction time, static balance, mobility
Boa Sorte Silva et al. [29]	67.5 ± 7.3	In person	3x week 45 min multiple-modality exercise	24	VO_2_max, carotid arterial compliance, intima-media thickness, blood pressure
Boa Sorte Silva et al. [32]	67.5 ± 7.3	In person	3x week 45 min multiple-modality exercise	24	Spatiotemporal gait parameters, usual and dual task walking gait velocity, step length, cycle time variability
Boa Sorte Silva et al. [30]	67.5 ± 7.3	In person	3x week 45 min multiple-modality exercise	24	Cambridge Brain Sciences (CBS) and its subtests
Shellington et al. [52]	68.5 ± 6.0	In person	2 x week 60 min session (5–10-minute warmup and 5-to minute cooldown)	24	Feasibility, recruitment, attendance, four cognitive domains, memory, concentration, planning and reasoning, global cognitive function
Shellington et al. [53]	69.5 ± 7.4	In person	2 x week	24	Feasibility, recruitment, Knee OA symptoms, static and dynamic balance, mobility, fitness, walking speed
Sebastião et al. [50]	64.3 ± 4.5	Home-based (hybrid)	2–5 x week 10-15-to-25–30-minute sessions (progressing sessions)	12	Feasibility metrics: process, resource, management and scientific, T25FW,6 MW, TUG, SDMT, BVMT, CVLT, SPPB
Boa Sorte Silva et al. [31]	67.5 ± 7.3	In person	3x week 45 min multiple-modality exercise	24	Memory, task-related cortical and subcortical functional connectivity changes
Sawasdee et al. [48]	69.2 ± 4.2	In person	3 x week 30 min session	12	Heart rate, body fat
Uchida et al. [61]	78.7 ± 3.8	In person	1 session (3 targeted step patterns) with 160 s break between step pattern	Acute (1 single visit)	Heart rate, RPE, intensity.
Kim et al. [42]	70.3 ± 4.7	In person	1 x week 30 to 40 min sessions (10 min warm-up, 25 min SSE, 30 min WBE and 10 min cooldown)	20	physical activity, amino acid concentrations
Kocaman et al. [41]	77.6 ± 6.1	Home-based	3 x week	8	Composite balance score, sensory organization test, Montreal cognitive assessment, adaptation test, fall efficacy scale, vestibular disorders activities of daily life scale
Sharma et al. [49]	69.5 ± 5.0	In person	3x week 60 min session	8	General practitioner assessment of cognition scale
Soma et al. [28]	72.0 (69–78)	In person	-	-	SSE, Silver rehabilitation taisou exercise, distance from home, travel mode, interpersonal social network
Sow et al. [59]	73.0 ± 3.8	In person	3x week 60 min session (+ 10 min warm-up/10 min cooldown)	12	Retention, attendance, feasibility trail making test om executive function, gait
Kraiwong et al. [43]	71.5 ± 5.4	In person	3 x week 45 to 60 min session (10 min warmup, 25 min of SSE, 10–15 min RT, 10 min cooldown)	8	TUG, AST, FTSST, Montreal cognitive assessment, ADL
Liu et al. [45]	70.9 ± 7.1	In person	2x week 60 min session (+ 10 min warm-up/10 min cooldown)	8	Executive function, global cognition, quality of life, Montreal cognitive assessment
Cha et al. [37]	74.8 ± 6.7	In person	2x week 50 min session (+ 10 min warm-up/10 min cooldown)	12	Effect of SSE on fall related physical strength and BDNF and IGF-1
Lees et al. [44]	70.8 (65–76)	Home-based	10-min bouts to try to reach the goal of 150 min a week	3	Physical activity level, exercise barriers
Khalaji et al. [40]	67.1 ± 3.6	In person	Acute bout with 12-trials	1	Retention, transferability
Sadeghian et al. [47]	65.2 ± 3.82	In person	3x week 40–60 min session (+ 10 min warm-up/10 min cooldown)	8	Static and dynamic balance

Note: SSE – Square-Stepping Exercise; MMSE – Mini-Mental State Examination; GDS-15 – Geriatric depression scale short form; Knee OA – Osteoarthritis; TUG – Time up and go; CBS – Cambridge Brain Sciences; STS – sit to stand test; VO_2_max– Maximum oxygen consumption; RPE – rate of perceived exertion; T25FW – Timed 25-foot walking; 6 MW – 6-minute walk test; SDMT – Symbol digit modalities test; BVMT – Brief visuospatial memory test; CVLT – California verbal learning test; SPPB – Short physical performance battery; AST – Alternate stepping test; FTSST – Five-times-sit-to-stand test; HGS – Handgrip strength; ADL – Activities of daily living; BDNF – Brain-derived neurotrophic factor; IGF-1 – Insulin-like growth factor 1

### Summary of the SSE intervention findings

Table 3 displays the main findings regarding the SSE interventions in both apparently healthy older adults and older adults with neurological conditions. The predominant focus of the studies was on physical function, with 53% of studies (*n* = 20) concentrating on various parameters associated with physical function outcomes such as strength, balance, and functional fitness. Cognitive function was the primary focus of four studies [30, 31, 33, 36], in which the authors examined executive control, global cognition, and memory. Of the four studies in older adults with neurological conditions [50, 60, 63, 64], two studies [50, 60] observed a positive impact of the SSE on the assessed metrics of both physical and cognitive function in the analyzed populations (i.e., multiple sclerosis and stroke). The other two studies observed a positive impact of SSE in improving balance and gait in their Parkinson’s disease sample [63]; and while the other observe that SSE was effective in improve cognitive function in their sample of Parkinson’s disease patients [64]. Among the 37 studies, 32 (84%) demonstrated significant improvements in the selected outcomes, whether related to physical function, cognition, or both. Notably, one study comparing two groups (multi-modality exercise plus SSE as intervention versus multi-modality plus balance, range of motion and breathing exercises as a control group) reported that adding SSE to a multi-modality exercise program was not effective to improve mobility outcomes when compared to the described control group [32]. Feasibility was the focus of 11% (*n* = 4) studies [50–52, 59], while one study [28] delved into the nuances of participation and delivery within an exercise program. All the studies showed that SSE was feasible and safe for the assessed population.

**Table 3:** Main findings observed in the 37 included studies on the interventions involving square-stepping exercise

Author	Summary of the findings
Shigematsu et al. [19]	SSE was effective in improving lower-extremity functional fitness.
Shigematsu et al. [54]	SSE is safe and acceptable for older adults, and it improves the functional fitness of the lower extremities.
Shigematsu et al. [56]	SSE is safe, acceptable and equally effective as a strength and balance training to improve functional fitness.
Shigematsu et al. [55]	SSE participants did not report adverse events such as falling, severe muscle soreness or dizziness when compared to a walking group.
Teixeira et al. [57]	SSE training, as a global cognitive stimulation, has positive influence on cognition in older adults, particularly on concentrated attention, mental flexibility, and visual memory.
Teixeira et al. [58]	Participants who engaged in basic physical exercises improved in agility and aerobic endurance, and SSE improved balance tests.
Pereira et al. [46]	SSE showed improvements in GDS-15 scores and on the time to perform the TUG test when compared to the control group after intervention.
Tubero et al. [60]	SSE improved dynamic balance and cognition in patients with cerebrovascular accident compared to physiotherapy group.
Gill et al. [36]	Dual tasking exercise group showed improvements in global cognitive function compared to exercise only.
Gregory et al. [35]	Dual tasking exercise (SSE) improved gait performance but not vascular health in older adults compared to a motor exercise only.
Jindo et al. [38]	SSE with goal setting improved the physical activity levels of the participants compared to the group without goal setting.
Jindo et al. [39]	Providing a pedometer during an exercise intervention is an effective addition to improve lower-extremity physical function.
Jindo et al. [62]	Participants engaged in higher levels of physical activity experienced grater improvement in lower-extremity function from the exercise program compared to those with lower daily activity levels.
Ravichandran et al. [63]	SSE is more effective as compared to CPT program in improving balance and gait in Parkinson’s disease patients.
Heath et al. [33]	Improvements in oculomotor processing in persons with self-reported cognitive complaint following 24-week multiple-modality training program.
Shellington et al. [51]	The HealtheBrain app was feasible in providing SSE to older adults.
Chang et al. [34]	A combination of aerobics and selected fall-prevention exercises (SSE) performed over a consistent period may improve mobility without compromising the fundamental benefits of aerobics.
Boa Sorte Silva et al. [29]	Mind-motor training (SSE) associated with multiple-modality exercise can positively impact cardiovascular fitness.
Boa Sorte Silva et al. [32]	SSE program improved aspects of lower-extremity physical function that require complex physical performance.
Boa Sorte Silva et al. [30]	Additional mind-motor training (SSE) was not effective to improve mobility outcomes, participants in the active control group presented greater benefits.
Shellington et al. [52]	Improvements in cognition in individuals with subjective cognitive complaints.
Shellington et al. [53]	Improvements in cognitive function, specifically executive control after intervention.
Sebastião et al. [50]	SSE presents limited feasibility with knee osteoarthritis. However, shows a trend to improvements in lower extremity mobility and walking speed.
Boa Sorte Silva et al. [31]	SSE-MS intervention improved physical and cognitive function, it is safe and feasible.
Sawasdee et al. [48]	Multiple modality exercise with mind–motor training resulted in greater improvements in memory compared to an active control group.
Uchida et al. [61]	Modified SSE for 12 weeks was sufficient to reduced body fat of the intervention group.
Kim et al. [42]	Body weight, body mass index, body mass, household physical activity, total physical activity, tyrosine, and phenylalanine significantly increased after 20 weeks in the Ex + CIT LEU group.
Kocaman et al. [41]	Posturography balance exercise and SSE training was more effective to improve functionality than vestibular training in older fallers.
Sharma et al. [49]	Cognitive training and mind motor training where equally significant beneficial in improving cognitive and functional skills.
Soma et al. [28]	Women’s participation in preventive care exercises (silver-rehabili taisou exercise and SSE) was negatively associated with a longer distance to a facility.
Sow et al. [59]	SSE is feasible and has a positive effect on executive function and gait variability.
Kraiwong et al. [43]	SSE intervention was effective to improve balance and lower limb muscle strength among older adults with type 2 diabetes mellitus and balance impairment.
Liu et al. [45]	SSE provided positive and significant effects on cognitive function, especially executive function in individuals with Parkinson’s disease.
Cha et al. [37]	The SSE program had a positively effect on fall-related fitness and BDNF levels.
Lees et al. [44]	SSE is an intervention that helps overcome barriers such as being uncomfortable in a fitness facility and body image and activate enablers such as the use of home equipment and convenience.
Khalaji et al. [40]	Incorporation of motivational and attentional factors can further enhance the effectiveness of training on this task relative to the typical standardized practice.
Sadeghian et al. [47]	SSE and Taichi are both effective to improve functional fitness and reduce fear of falling.

Note: SSE -Square-Stepping Exercise; GDS-15 – Geriatric depression scale short form; Knee OA – Osteoarthritis; TUG – Time up and go; CVA – cerebrovascular accident; BDNF – Brain-derived neurotrophic factor; GDS-15 – Geriatric depression scale short form; TUG – Time up and go; CIT- citrulline; LEU – leucine

## Discussion

The present scoping review provided an overview of the protocols and outcomes of studies employing SSE in older adults. Our findings indicated that SSE has been used as an exercise strategy to improve various functional fitness, and physical and cognitive function parameters among both apparently healthy older adults and older adults with neurological conditions (i.e., Parkinson’s disease, multiple sclerosis, and stroke) in different parts of the globe. The studies employing SSE involved a varied of protocols in terms of frequency, session duration, intervention length, and delivery mode. Collectively, the findings from the included studies demonstrated the effectiveness of SSE as intervention, frequently yielding positive improvements in various clinical and non-clinical health parameters of older adults.

Physical activity, particularly exercise training, has been highly recommended for older adults, even in the presence of chronic diseases to improve general health and well-being, and to maintain and/or improve function [14]. The SSE studies described in the 37 research articles included in this review demonstrated considerable variability. Despite the variability, the studies adopting intervention demonstrated alignment with current exercise recommendations - regarding frequency and a duration - for both populations of older adults [6, 16]. For instance, the average frequency and duration of the session observed in the studies were three sessions per week with duration of 90 min. Current exercise recommendations for older adults recognize the multifaceted nature of aging, emphasizing various physical capacities such as strength, flexibility, balance, and cardiorespiratory fitness [6, 16]. While the guidelines underscore specific types of physical activities, they also advocate for a minimum of 150 min of moderate-intensity or 75 min of vigorous-intensity exercise weekly [6, 16]. SSE aligns with these recommendations, offering a type of exercise that not only satisfies the diverse physical capacities but also may assist older adults in achieving current physical activity guidelines [6]. Further, current exercise guidelines tailored for neurological patients (i.e., Parkinson’s disease, multiple sclerosis, and stroke) recommend a frequency ranging from 2 to 3 days per week with session durations spanning from 10 to 60 min [5]. Exercise guidelines for neurological diseases underscore the necessity of an individualized approach, acknowledging the necessities and capabilities of each subject, providing a safe range for exercise prescription, and highlighting progression. SSE aligns with the exercise guidelines for this population in that the program offers different levels of step routine complexity (i.e., beginner, intermediate, advanced) making it easy to individualize the program according to the capabilities of each older adult patient.

The observed diversity in delivery forms of SSE is worth discussing. Studies employed three primary delivery modes: online, in-person, and at-home. While in-person sessions emerged as the most prevalent, this review highlighted the feasibility and positive outcomes associated with all delivery methods, including a hybrid/home-based approach conducted in older adults with multiple sclerosis [50]. Among the three delivery methods, the most employed form was in-person. The in-person method of delivery may offer several advantages, including supervision, motivation, and social interactions. On the other hand, the two alternative delivery methods, home-based and online, present the advantage of being done at flexible hours, at home, potentially overcoming barriers related to exercise program accessibility. However, these two methods (home-based and online) exclude social interaction and proper supervision, which may be important factors to ensure that the exercise intervention is done properly for certain populations. Nevertheless, the lack of these elements may not necessarily hinder the effectiveness of the intervention. In this review, five articles [41, 44, 50, 51] employed a home-based or hybrid delivery approach. For instance, Fawzy et al. [65] showed that either home-based or supervised (in person) delivery methods significantly improved balance performance in older adults. However, Ashworth et al. [66] demonstrated that home-based interventions exhibit better long-term adherence compared to supervised activities, which, on the other hand, show higher short-term results. Collectively, these findings highlight that different delivery methods have their strengths and limitations.

The SSE was created with the focus of developing an intervention specifically designed to improve lower-extremity function and therefore reduce the risk of falls among older adults [19]. The unique design of the SSE aims to comprehensively address various physical capacities highlighted in exercise guidelines tailored for the older adult population, encompassing muscular strength, balance, flexibility, and cardiorespiratory capacity [6, 16]. The complex patterns and the high cognitive demand observed in SSE demonstrate that such an intervention has the potential to have a positive impact on both the physical and cognitive function of older adults. Such a dual focus contributes to the overall well-being of older adults, both from the general population and those presenting with neurological diseases, by simultaneously targeting key parameters highly associated with health and independence. The evolution of SSE from a targeted physical intervention to a comprehensive approach underscores its adaptability and the recognition of its potential benefits beyond the original scope. Its simplicity, safety, and adaptability to home use make it a valuable form of exercise for older adults with or without neurological conditions. Overall, the exercise’s low-to-moderate intensity [61], coupled with its adaptability for home use, makes it a practical, viable, and effective exercise choice for apparently healthy older adults and those with neurological conditions resulting in both physical and cognitive impairment.

Importantly, despite generally yielding improvements in the selected studies’ outcomes, significant variability was observed in the SSE studies regarding study design, intervention protocols, outcomes, and participant characteristics (see Tables 1 and 2, and 3). Such methodological differences could partly explain the discrepancies in the magnitude of improvements and/or possibly conflicting results observed among the studies. For instance, some studies combined SSE with other exercise modalities, such as yoga, tai chi, and multi-component exercises, leading to diverse protocols. This makes direct comparison challenging, as it is difficult to isolate the effects of SSE alone. This is exacerbated by the fact that large variability in terms of frequency, duration of the exercise session, and length of the intervention was also observed. Moreover, SSE includes over 200 different step patterns with varying complexity (beginner, intermediate, advanced). The selection of step patterns and complexity levels varied significantly based on participants’ physical and cognitive abilities. For example, some studies were conducted with apparently healthy older adults, while others recruited older adults with neurological conditions (e.g., Parkinson’s disease, stroke, multiple sclerosis) known to directly impact physical and cognitive function. Additionally, although the studies primarily focused on physical and cognitive function outcomes, they used a wide range of specific measures within these broad categories.

### Limitations

This scoping review, while providing valuable insights, presents limitations that highlight potential opportunities for future studies. The use of only five databases during the search and selection process, combined with the restriction to articles in English, may introduce language and database biases, potentially excluding relevant studies in other languages. SSE was originally tailored for older adults; therefore, the focus of this review is understandably limited to this demographic. While this facilitates targeted exploration, it challenges the generalization of SSE’s efficacy to younger age groups.

We found only four studies that explored the effects of SSE on physical and/or cognitive outcomes in older adults with neurological conditions such as multiple sclerosis, Parkinson’s disease, and stroke. No studies explored the potential of SSE in older adults with other neurological conditions, such as mild cognitive impairment, early-stage dementia, or epilepsy. This underscores the potential for future studies to explore the applicability of SSE in other neurological contexts. Additionally, no study has compared SSE with established mind-body/physical-cognitive exercise modalities such as Tai Chi and yoga. Furthermore, only three studies have investigated SSE in combination with other exercise modalities, highlighting the need for additional evidence.

Considering the potential combined benefits of different exercise modalities, future studies should focus on developing and testing interventions that integrate SSE with other exercise modalities while also examining a broader range of physical, psychological, and social outcomes. Out of the 37 included studies, only four employed SSE in combination with traditional exercise modalities (i.e., resistance training and aerobic). Nevertheless, to the best of our knowledge, this is the first review mapping the scope of SSE in older adults. This study provides a comprehensive synthesis of the existing literature on SSE that can assist in the development of future studies employing SSE in both healthy and diseased populations of older adults.

To address the above-described gaps, future studies adopting SSE should:

- Extend the SSE research to include populations with common neurological conditions (e.g., mild cognitive impairment, early-stage dementia). These studies could aim to understand the specific benefits and adaptations needed for SSE in these groups, potentially including mechanistic studies involving biomarkers and advanced neuroimaging techniques.
- Conduct head-to-head comparisons that directly compare SSE with other well-established non-traditional modalities such as Tai Chi and yoga. These studies could adopt a crossover design where participants engage in each type of exercise modality in different phases or a parallel group design, where each participant is assigned to one exercise modality for the entire study duration.
- Conduct RCTs to compare the effects of SSE alone, traditional exercise modalities (e.g., aerobic or resistance training), and combined interventions (e.g., SSE plus resistance training) on comprehensive health outcomes, including physiological markers, physical and cognitive function, emotional well-being, and social participation and autonomy.
- Develop and test multi-component exercise programs that integrate SSE with other modalities (e.g., combining SSE with balance training and stretching exercises) and examine their effects on comprehensive and important health-related outcomes, including but not limited to risk of falls.
- Implement longitudinal studies to investigate the long-term benefits of combined SSE and traditional/non-traditional exercise modalities and assess sustained impacts on health-related quality of life, physical and cognitive function, independence, and reduction of fall risk over time.

By addressing these areas, future research can provide more robust evidence on the possibilities and efficacy of SSE, explore potential synergistic effects with other exercise modalities, and ultimately enhance the physical, cognitive, and overall health and well-being of older adults.

## Conclusion

This review comprehensively described the characteristics of 37 studies employing SSE in apparently healthy older adults and older adults with neurological diseases. The findings demonstrated that SSE has been used by researchers across the globe as a viable exercise modality, particularly to improve functional fitness, and physical and cognitive function of different segments of the older adult population. In addition, it was observed that SSE has been effective regardless the form of delivery (i.e., in-person, online, hybrid/home) or protocol adopted, with most of the studies generally following current exercise recommendations for older adults as far as frequency and duration. Most of the included studies focused on healthy older adults, with reported outcomes primarily in the domains of physical function, followed by cognitive function. However, gaps were identified, including a limited range of outcomes assessed, a lack of research on the combination of SSE with traditional and non-traditional exercise modalities to understand potential synergistic effects, and the use of SSE for populations with common neurological conditions (e.g., mild cognitive impairment, dementia). Thus, future research should prioritize the inclusion of comprehensive outcome measures, direct comparisons between SSE and other non-traditional modalities, and the adoption of integrated interventions with rigorous methodological approaches. This will provide high-quality evidence on the efficacy and feasibility of SSE in combination with other exercise modalities. Additionally, SSE research should be extended to specific populations with neurological conditions, ensuring that interventions are tailored to their unique needs and capabilities. This scoping review described and analyzed important aspects related to SSE programs as well as main outcomes assessed in apparently healthy older adults and those with neurological diseases. The information generated by this study may serve as basis for future studies attempting to employ SSE as standalone intervention or in combination with other traditional and non-traditional forms of exercise (e.g., resistance training, yoga) to improve important health-related parameters among these populations. The adaptability of SSE to be combined with other exercise modalities, used across diverse older adult populations, and delivered through various modes (e.g., in-person, online, hybrid) underscores its democratic nature. This flexibility positions SSE as potential exercise option for health professionals engaged in the care of older adults, facilitating the promotion of physical activity and exercise among older adults with and without neurological conditions.

### Electronic supplementary material

Below is the link to the electronic supplementary material.

Supplementary Material 1

## Data Availability

All data generated or analyzed during this study are included in this published article and are available from the corresponding author upon request.
