# Supplementary figures and images for: Stepping towards health: a scoping review of square-stepping exercise protocols and outcomes in older adults
Source: BMC Geriatr. 2024 Jul 10;24:590. doi: 10.1186/s12877-024-05187-8 (PMC11238358; doi:10.1186/s12877-024-05187-8)

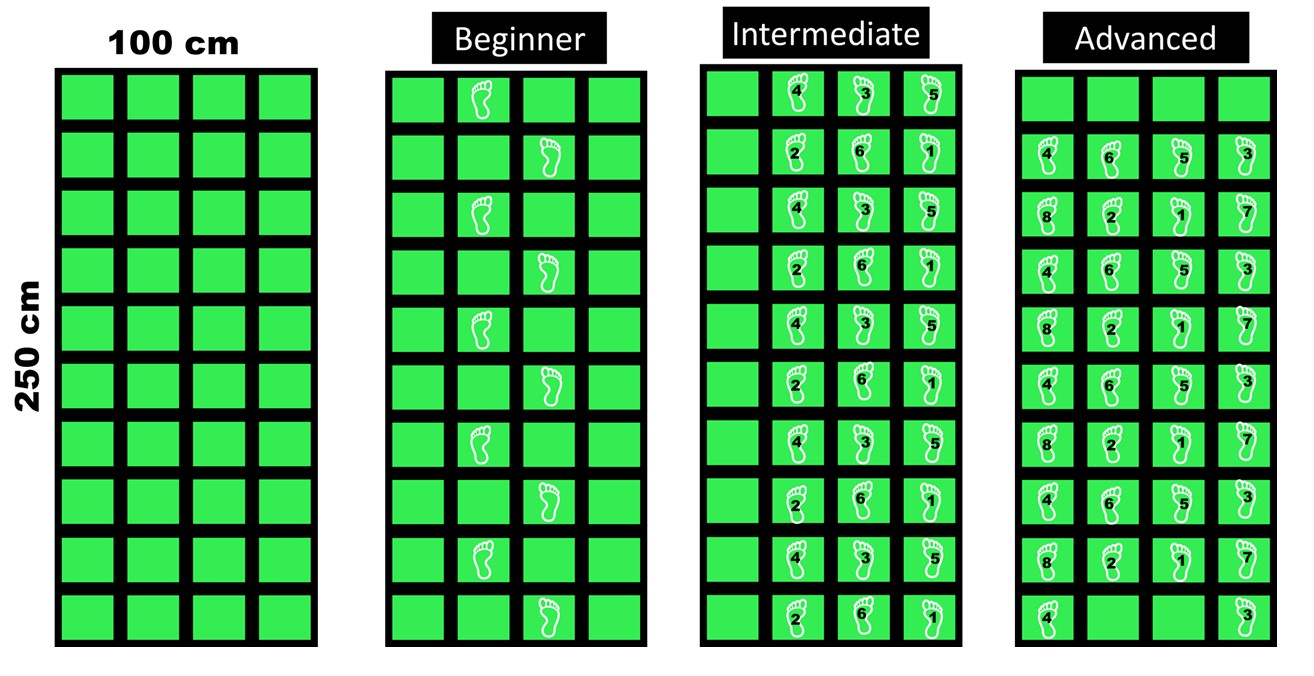

Supplement: Supplementary file 1 — Supplementary Material 1 [file 12877_2024_5187_MOESM1_ESM.jpeg]
